# A Novel Conservation Genomic Strategy: Selection for the Probability of Offspring Heterozygosity

**DOI:** 10.3390/ani15152217

**Published:** 2025-07-28

**Authors:** Attila Zsolnai, András Nagy, Gábor Szalai, Ino Curik, István Anton, Péter Hudák, László Varga

**Affiliations:** 1 Department of Animal Breeding, Institute of Animal Science, Hungarian University of Agriculture and Life Sciences, Kaposvár Campus, 7400 Kaposvár, Hungary or icurik@agr.hr (I.C.); anton.istvan@uni-mate.hu (I.A.); 2 Lunenfeld-Tanenbaum Research Institute, Mount Sinai Hospital, Toronto, ON M5G 1X5, Canada; nagy@lunenfeld.ca; 3 Australian Regenerative Medicine Institute, Monash University, Clayton 3800, Australia; 4 Department of Obstetrics and Gynecology, University of Toronto, Toronto, ON M5G 1E2, Canada; 5 Institute of Medical Science, University of Toronto, Toronto, ON M5S 1A8, Canada; 6 Department of Biomedical Sciences, Burrell College of Osteopathic Medicine, 3501 Arrowhead Drive, Las Cruces, NM 88001, USA; gszalai@burrell.edu; 7 Department of Animal Science, Faculty of Agriculture, University of Zagreb, Svetošimunska Cesta 25, 10000 Zagreb, Croatia; 8 Institute for Farm Animal Gene Conservation, National Centre for Biodiversity and Gene Conservation, 2100 Gödöllő, Hungary; hudak.peter@nbgk.hu; 9 Institute of Genetics and Biotechnology, Hungarian University of Agriculture and Life Sciences, Szent István Campus, 2100 Gödöllő, Hungary; varga.laszlo.andras@uni-mate.hu

**Keywords:** conservation, genetic diversity, genetic management, inbreeding avoidance, SNP

## Abstract

The primary objective of any conservation breeding program is to preserve the genetic diversity of populations. This objective is a persistent challenge, especially in small populations which are prone to loss of heterozygosity. In this study, we proposed a novel parent-selection strategy aimed at the long-term maintenance of high levels of genetic diversity. Our approach is based on estimating the Probability of Offspring Heterozygosity (POH)—the likelihood that a mating will produce heterozygous offspring—using SNP genotype data. This strategy was evaluated through computer simulations, where parental pairs with the highest POH values were preferentially selected to produce the next generation. Simulations explored the effects of varying the number of breeding pairs, and the number of unlinked SNP markers. Selection based on POH resulted in observed heterozygosity (H_OBS_) consistently exceeding expected heterozygosity (H_EXP_), a trend that was sustained for up to 1000 generations. While further evaluation is needed within more complex population genetic frameworks—accounting for linkage disequilibrium, recombination, optimal contribution, and phenotypic selection—our findings highlight the potential of POH as a valuable tool for enhancing genetic diversity in conservation breeding programs.

## Simple Summary

This manuscript introduces a proof of concept, a DNA-based, conservation management strategy that has demonstrated significant effectiveness in maintaining genetic diversity by identifying the combinations of parents that are most likely to produce highly heterozygous offspring. Our simplified experimental model reveals that both the observed and the expected heterozygosity can be kept at a high level with ten breeding pairs. This conservation genomic approach has the potential to prevent or reverse the extinction processes affecting endangered populations, breeds, and species, addressing one of the most pressing challenges in conservation studies.

## 1. Introduction

Genetic diversity is the genetic variation found in the genome of a species. Its loss makes species and populations vulnerable and increases their risk of extinction, so maintaining diversity is one of the most challenging task in small populations. Maintaining genetic diversity is a primary objective not only in conservation programs, but also in the management of different livestock breeds and in wild populations, especially of those having small population sizes. Genetic diversity management is a process that starts with a base population, whose diversity is the reference point for the diversity of the subsequent, managed generations. The level of genetic diversity is inherently determined by the size of the initial base population and by the individual genetic diversity of the members of that population. If the population size is small, the effective population size is typically even smaller, potentially leading to genetic drift, increased inbreeding, and homozygosity of deleterious recessive alleles. Such a population gradually loses its adaptive flexibility, and selection loses its genetic basis [1]. If genetic diversity cannot be maintained in such endangered populations in the long run, they might eventually become extinct. Consequently, diversity management is a priority in conservation programs, to sustain the maximal possible heterozygosity [2].

In the pre-genomic era, microsatellites were used to assess genetic variation [3], while, more recently, single nucleotide polymorphisms (SNPs) have become the predominant genetic markers due to their abundance in the genome and also the relative ease of large-scale genotyping using SNP-chips [4]. Different methods were suggested for managing the average kinship (i.e., coancestry [5]). These procedures involve two steps: the first is to determine the parental contributions, and the second is to define the mating strategy. The contribution and mating can be decided simultaneously in a single step (mate selection procedure) or separately in two steps, almost with the same efficiency [6]. Four methods were tested by computer simulations with various parameter settings for minimizing the average kinship in captive populations: the Static, Dynamic, Ranked and Simultaneous Mean Kinship Selection [5]. Until the advent of modern genetic markers, only genealogical approaches based on pedigree records were used for managing genetic diversity. Wright [7] proposed the method called Maximum Avoidance of Inbreeding, where theoretically, the least related individuals were mated, according to a predefined scheme.

Computer simulations were used to compare different conservation strategies, with the aim of maximizing genetic diversity. Random-mated base populations were simulated for a variable number of generations (e.g., 5 and 10) using different population sizes (e.g., N = 20, N = 100). The genomes considered were also varied with chromosome numbers 1 or 20, as well as the number of markers on the chromosomes (e.g., 100, 1000 and 10,000). Recombination events were also simulated in generating gametes with random occurrence on each chromosome [2,4,8].

The census size of the managed populations was also a variable, just like the number of the managed generations (e.g., 15; 10), when different management procedures were tested and compared. The first strategy acts on parents, optimizing their contribution (how many offspring they leave to the subsequent generation) based on their marker genotypes, thereby minimizing the expected coancestry. The other strategy acts on the offspring. In this procedure, the parents are mated at random, producing the same number of offspring (for example, four male and four female) [4], which are genotyped; those kept and used as parents of the following, third generation, have the minimal average observed coancestry [4,8].

Genetic diversity is usually measured either by the observed (H_OBS_), or by the expected (H_EXP_) heterozygosity, or by the allelic diversity (AD). H_OBS_ is the proportion of heterozygous genotypes for all the tested loci. H_EXP_ is the probability that two randomly chosen alleles from the population would be different. Allelic richness (AD) is the mean number of alleles over the range of examined loci [9,10]. Regarding heterozygosity, (H_OBS_, H_EXP_) with respect to different parent and offspring management strategies, the works from the above authors [3,4,8] led to several conclusions. When only parental genotypic information was used in the management procedure, where 10 or 100 markers were considered on the chromosome, results were slightly better than the minimum pedigree coancestry. Considerable performance improvement was detected when offspring data was used not only for eight, but even for two offspring [8]. Molecular information proved to be inferior to the genealogical information [3,4]. The offspring-based management using observed molecular data (H_OBS_) performed better than the strategy acting on the parents because the former maintains larger diversity than the latter, which proceeds with expected data (H_EXP_). The offspring-based strategy is highly applicable in conservation schemes of multiparous species, where the manager has to select individuals for further breeding [4]. Another study emphasizes that molecular coancestry outperforms genealogical coancestry only if the SNP chip marker density (SNP/Morgan) is high, at least 3 times the effective population size [2]. Several molecular coancestry measurements have been reported [11,12,13], and their efficiency and effects on population characteristics were compared in detail [1,14,15]. Coancestry measurements and their applications in selection programs focus on the parental generation determining the degree of relationship or similarity between the mating pairs, using this information to minimize the loss of heterozygosity.

Here, we describe an approach which uses the offspring’s probability of heterozygosity, a shift from using the number of shared alleles [13]. The concept of our model is to select specific breeding pairs from all mating combinations that can theoretically produce litters expected with the highest overall heterozygosity value, termed Probability of the Offspring Heterozygosity (POH), where the POH for a locus is calculated by assuming Mendelian segregation of a biallelic marker (for details, see Section 2.4, Calculation of the POH value). Our aim was to investigate the applicability of POH selection in its pure form using a reduced set of unlinked molecular markers when selection for genetic gain and use of different relationship matrices [1,16] were not considered. The starting point of our simulations is a real base population with known SNP genotypes [17]. The results of POH selection were compared to those of a proportion (ASp) derived from the number of shared alleles [13].

In our work, we examined how the initial H_OBS_ and H_EXP_ values of a real base population vary over the subsequent simulated generations employing POH selection. Different parameter combinations were used with respect to the SNP number, the number of breeding pairs and the simulated generations. Since it is not possible to show all combinations of all the parameters, we present some typical sets and runs, which give insights into how the model works.

Mating pairs with the highest POH values were selected to produce the next generation. The same selection step is repeated for each subsequent generation, identifying the best mating pairs. POH values based on the mating type are given in Section 2.4. In the models, generation overlapping, linked markers, and recombinations were not implemented. Recombinations were only considered when the fate of the loci under no POH selection was investigated.

## 2. Materials and Methods

### 2.1. Base Population

The base population consisted of 70 Hungarian Short-haired Vizsla (HSV) dogs in a ratio of 29 males to 41 females [17]. The animals were selected to represent most of the lines of the Vizsla population. Trained veterinarians collected 5-milliliter blood samples from pedigree-certified individuals into EDTA-coated tubes, which were subsequently stored at −20 °C as part of a routine procedure for parentage testing; therefore, no ethical approval was required.

### 2.2. SNP-Chip/Markers

SNP typing of the samples was performed on Illumina Canine HD chip containing 234k SNPs (Illumina, San Diego, CA, USA) by Neogen Corporation (Ayr, UK). After quality check, call rate was set to exceed 0.95, and mitochondrial, X and Y chromosomes were excluded, leading to 206,267 informative SNPs. Genotypes were converted to raw format where the two forms of homozygous animals were 0 (AA) or 2 (BB), while the heterozygous animals were recoded as 1 (AB).

### 2.3. SNP-Set for Calculations

For simulations, SNPs inherited independently and displaying MAF (Minor Allele Frequency) value greater than 0.4 were used. The filters on the call rate of markers and that of the samples were set to 1. From the 206,267 SNP-chip set (see Section 2.2) 28,806 biallelic SNPs had MAF > 0.4. Calculations were performed with 1, 2 or 3 markers, and with an experimental, reduced whole-genome set (51 SNPs) where all the independently inherited regions of the genome were represented only by a single SNP. These unlinked regions were determined according to the cM data of the latest Comprehensive Canine Linkage Map [18] constructed from 3000 microsatellites. For chromosomes longer than 50 cM, proximal and distal regions of independence were defined on both sides of the centralized 50 cM separation zone. The Mbp coordinates corresponding to these two cM positions were defined on the basis of the Comprehensive Canine Linkage Map [18]. The representative SNPs were selected from the above-described 28,806 SNPs. The same procedure was performed for chromosomes below, or slightly above, 50 cM (CFA14-CFA38), as well. For chromosomes shorter than 50 cM, one SNP was selected from the central region. A total of 51 unlinked SNPs were selected, serving as a minimal marker set for the current experimental simulations, listed in Table 1.

### 2.4. Calculation of the POH Value

**Figure 1:**
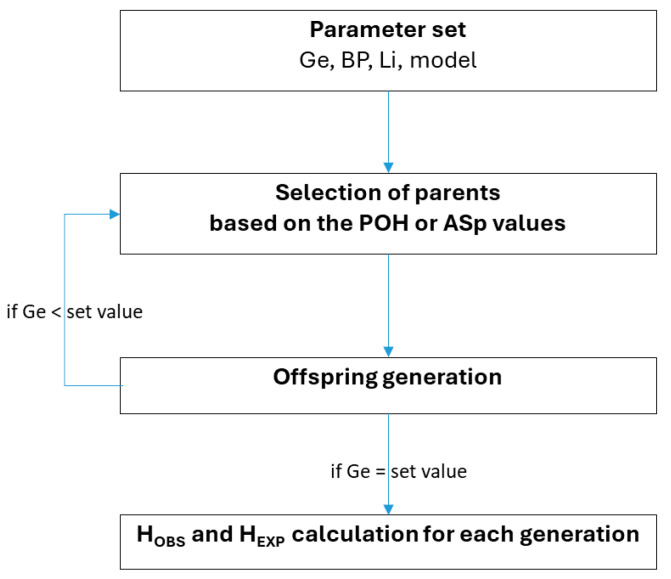
The scheme of POH selection. Ge: the number of simulated generations, BP: the number of breeding pairs, Li: the number of offspring per litter, POH: Probability of Offspring Heterozygosity, Asp: proportion of shared alleles, H_OBS_: observed heterozygosity, H_EXP_: expected heterozygosity.

The POH value is calculated from the individual genotypes of all parental male–female pairs. Littermates were excluded from being a breeding pair. For a single locus, the POH value is defined as 1 when both parents are homozygous but for different alleles, and as zero when the parents are homozygous for the same allele, while it is defined as 0.5 when both parents are heterozygous or one parent is heterozygous and the other is homozygous (Table 2). For two or more unlinked loci, the probability values were averaged. The mating pairs with the highest POH values were selected to serve as the parents of the next generation (Figure 1). The generation of the genotypes of the descendants was based on Mendelian genetics. During the POH or ASp selection (see the next section), the POH or ASp values were the sole selection criteria of the parents, respectively. The H_OBS_ and H_EXP_ values were calculated to monitor the status of the subsequent generations. Fifty runs were performed for each set of identical parameter conditions. The simulations were performed in Linux environment, using Python 3.9.7 [19], with libraries numpy 1.21.2 [20], pandas 1.3.3 [21], matplotlib 3.4.3 [22], and ray 2.2.0 [23].

In the Pairs column, the digits 0, 1, and 2 represent a locus with homozygous genotype for allele1, heterozygous genotype, and homozygous genotype for allele2, respectively. The digit pairs 00, 22, 20, 02 stand for mating of homozygous animals; 11 is for mating of heterozygous animals, while 10, 01, 12, and 21 depict the mating of a homo- and a heterozygous individual. POH: Probability of the Offspring Heterozygosity. ASp: proportion of shared allele, ASp_(0–1)_: ASp values converted into 0–1 range (ASp_(0–1)_) to make comparison of POH-ASp values easier.

### 2.5. Calculation of Proportion of Shared Alleles

Proportion of shared alleles (ASp) between parents for a biallelic locus was calculated as ASp_ij_ = 0.25 * (number of shared alleles). The number of shared alleles [13] between animals i and j is two when both parents are homozygous for the same allele (00, 22, Table 2) or both are heterozygous (11), and one if a parent is homozygous and the other is heterozygous (10, 01, 12, 21). The number of shared alleles is zero in the case of mating of homozygous animals for the opposite alleles (20 or 02). When more than one locus is considered, the ASp values of the respective loci were averaged. In Table 2, the ASp values were converted to 0–1 range for better comparison with POH values. Please note that POH and ASp_(0–1)_ values take opposite or the same values for different mating types, and the only exception is in the case of heterozygous parents (Pair 11, Table 1). The mating pairs with the lowest ASp values were selected to serve as the parents of the next generation. Fifty runs were performed for each set of identical parameter conditions using the same libraries described in the previous section.

### 2.6. Random Selection

Random-mated simulation was performed similarly to POH and ASp selection, but the breeding pairs were selected randomly from each generation, and no selection criteria were applied. Fifty runs were performed for each set of identical parameter conditions.

### 2.7. Calculation of H_OBS_ Ad H_EXP_

As for H_OBS_ index for a given generation, first, the $n_{k}$ (the number of heterozygous animals at a locus *k*) was divided by the number of sampled animals (*N*). Observed heterozygosity of an individual (H_OBS_i_) was obtained to average the sum of $n_{k}/N$ across all loci (*L*), then H_OBS_ was acquired by averaging H_OBS_i_ values across all individuals.

$$H_{OBS\_i}=\frac{\sum_{k=1}^{L}\frac{n_{k}}{N}}{L}$$

$$H_{OBS}=\frac{\sum_{i=1}^{N}H_{OBS\_i}}{N}$$

As for H_EXP_ index for a given generation, first, the $2p_{k}q_{k}$ values (expected heterozygosity of an animal at locus *k*, where *p_k_* and *q_k_* is the frequencies of allele 1 and 2, respectively) were summed and averaged across all loci to obtain expected heterozygosity of an individual (H_EXP_i_), then H_EXP_ was acquired by averaging H_EXP_i_ values across all individuals.

$$H_{EXP\_i}=\frac{\sum_{k=1}^{L}2p_{k}q_{k}\;}{L}$$

$$H_{EXP}=\frac{\sum_{i=1}^{N}H_{exp\_i}}{N}$$

### 2.8. Parameter Conditions

Different experimental arrangements were tested. For better clarity, each parameter combination is described using a uniform abbreviation system, which is listed as ‘Parameter conditions’ at the beginning of the corresponding paragraphs.

The following parameters were adjusted before calculations:

M = Marker/SNP number = 1, 2, 3, 51.

BP = Number of breeding pairs = 3, 5, 7, 9, 10, 11, 13, 15, 17, 19, 21.

Li = Number of offspring per litter = 4.

Ge = Number of simulated generations = 50, 1000.

For example, M51-BP10-Li4-Ge50 is for Marker/SNP number = 51; Number of breeding pairs = 10; Number of offspring per litter = 4; Number of generations = 50. In that case, where the number of BP is 10 and the number of offspring is 4, there are 40 animals in the next generation.

Among the selected breeding pairs, an individual was only included once. The generations were not overlapping.

### 2.9. Data Visualization

**Figure 2:**
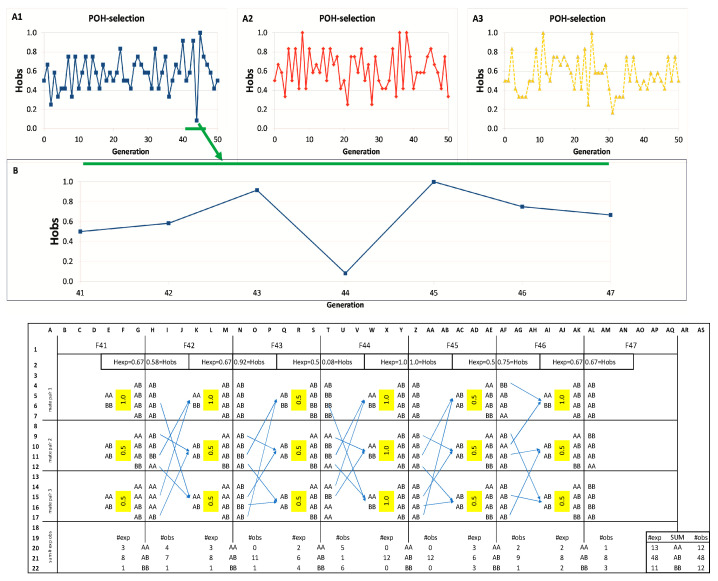
Simulations with one SNP. (**A1**–**A3**): plots of H_OBS_ values in POH selection. Different colors of lines represent different simulations. (**B**): Steps of simulation with 1 SNP at generations F41–47. The green arrow and the green horizontal bars represent the zoomed-in section of (**A1**). Generation: subsequent pools of offspring generated from the selected pairs of the previous population. Under the enlarged part of (**A1**), the selection steps for these generations at the individual level are shown. Yellow highlight is the POH value of the offsprings of a given pair. AA, AB, and BB: genotypes of a locus. #obs and #exp: observed and expected number of AA, AB, and BB individuals. Hobs: observed heterozygosity, Hexp: expected heterozygosity. H_EXP_ and H_OBS_ values are outlined by providing bold borderlines of the corresponding cells. This Figure is also in Figure S2, where the table can be seen in an Excel table.

In the case of Figure 2 and Figures S1–S4, we present typical runs of the simulations.

**Figure 3:**
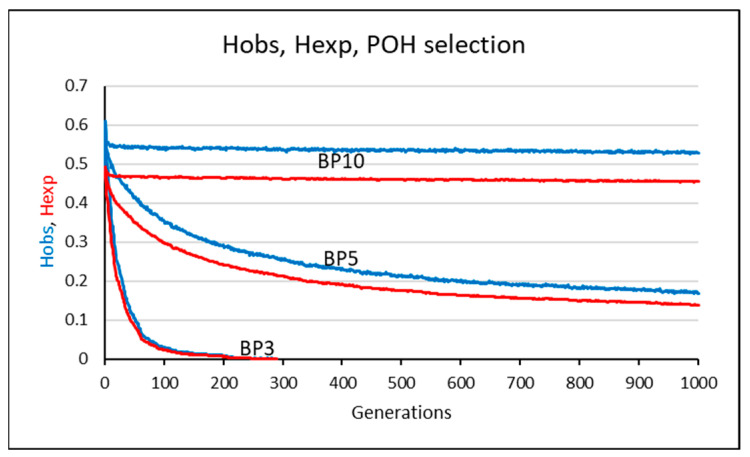
POH selection with 51 SNP, generation number 1000. Litter size is four. BP: number of breeding pairs (BP = 3, 5, and 10). Generation: subsequent pools of offspring generated from the selected pairs of the previous population. The observed and expected heterozygosity (Hobs, Hexp) are plotted with blue and red lines, respectively. Each line was obtained by averaging the values of 50 simulations.

**Figure 4:**
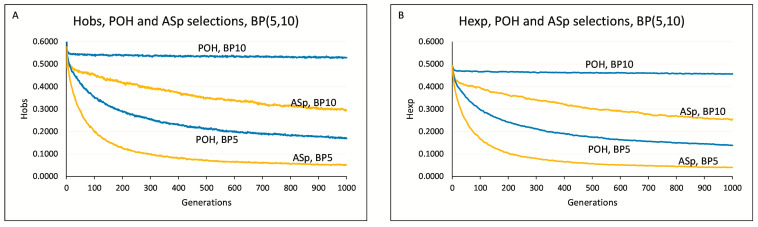
POH (blue line) and ASp (orange line) selection in the case of breeding pairs equal to 5 and 10. Generation number is 1000. Litter size is four. Generation: subsequent pools of offspring generated from the selected pairs of the previous population. Parts (**A**,**B**) are showing the observed and expected heterozygosity (Hobs, Hexp) of the same simulations, respectively. Each line was obtained by averaging the values of 50 simulations.

To reduce fluctuations from the numerous repetitions and generations, we averaged the H_OBS_ and H_EXP_ values across 50 runs for each set of identical parameter conditions. This approach, instead of showing individual graph lines, results in a more representative band in Figure 3 and Figure 4, and Supplementary Figure S5, effectively smoothing out fluctuations in the POH and ASp selections. In the case of Figure S3, averaging of observed heterozygosity in the subsequent generation was performed by pandas’ exponentially weighted moving average function [21] (H_OBS_n_smoothed_ = (H_OBS_n_ + (1 − α)H_OBS_n−1_ + (1 − α)^2^H_OBS_n−2_ + … + (1 − α)^N^H_OBS_n-N_)/(1 + (1 − α)+ (1 − α)^2^ + … + (1 − α)^N^), where n denotes a corresponding value at the nth position, and H_OBS_ is the observed heterozygosity. α is the smoothing factor, calculated as 2/(N + 1), where N is the number of data values spanned in a period.

## 3. Results

### 3.1. POH Simulations with One SNP (H_OBS_)

Parameter conditions: M1-BP3-Li4-Ge50. Three simulations of POH selection with a single marker are presented with real SNP genotypes in Figure 2(A1–A3). Simulations start from H_OBS_ = 0.485 value of the base population. All three simulations reached at least once the H_OBS_ = 1.0 value, representing 100% heterozygosity, as seen in Figure 2(A1–A3). However, these high values could not be maintained; they had uniformly fallen back in the following generation, and this skewing pattern has continued but never actually reached the H_OBS_ = 0 value, despite the small number of breeding pairs (BP = 3). The POH simulations never led to a complete loss of heterozygosity (not zeroed out), but the initial H_OBS_ value was maintained as a tendency over the 50 generations. The random models displayed wide oscillations and a trend to decrease the H_OBS_ value to zero eventually (Figure S1).

### 3.2. The Curve Fluctuations in the Selection Steps at the Individual Level

The most extensive alternation is observed at generations F43-44-45, where H_OBS_ shifts dramatically from 0.92 to 0.08 and then to 1.0 (visualized as the blue curve, Figure 2(A1)). To reveal how oscillation is created by the POH-selection model, generations F41-47 were inspected in more detail, as shown in Figure 2B.

At generation F41, three mating pairs were selected for breeding (column E), based on their high POH (1.0, 0.5, 0.5; Column F) producing the 12 individuals of F42 (Column H). For generation F43, breeders were chosen, now from F42 based on POH (1.0, 0.5, 0.5; Column L). This resulted in F43 having a high observed heterozygosity (H_OBS_ = 0.92; Cell N2), exceeding the expected value (H_EXP_ = 0.67; Cell M2), because the simulation generated a high proportion of heterozygotes (ten AB vs. two BB; Column N).

This high heterozygosity in F43 meant that the breeders selected for F44 (Column Q) were mostly heterozygotes (five AB, one BB). Consequently, the highest possible POH for these pairings was only 0.5 (Column R). Breeding these pairs led to generation F44 exhibiting a sharp decline in heterozygosity. The observed H_OBS_ dropped to just 0.08 (Cell T2), far below the expected H_EXP_ of 0.5 (Cell S2), because the simulation produced an excess of homozygotes (eleven in total: five AA and six BB; Column T).

The resulting overrepresentation of homozygotes in F44 provided the opportunity to select three AA × BB pairs for creating generation F45, maximizing the POH value at 1.0 for each pair (Column W). These matings predictably yielded 100% heterozygous offspring, causing both expected and observed heterozygosity (H_EXP_ and H_OBS_) to reach the maximum value of 1.0 (Cells Y2, Z2). From this peak heterozygosity, however, a decline is unavoidable. To create generation F46, only AB × AB pairs are available (Column AC), limiting the POH to 0.5 (Column AD) and setting the stage for reduced H_OBS_ in the next generation.

This cyclical pattern arises directly from the POH selection method. Seeking maximum heterozygosity can produce a generation composed entirely of heterozygotes (H_OBS_ = 1.0). Mating these individuals (AB × AB) inherently yields a mix of genotypes (AA, AB, BB), lowering H_OBS_ and H_EXP_, as well. The presence of both homozygote types then allows selection of high-POH pairs (AA × BB; POH = 1.0), which in turn boosts H_OBS_ in the following generation, maintaining the oscillation shown in Figure 2B.

The number of expected versus observed genotypes were compared (Figure 2B rows 20–22, Figure S2). Aggregating these counts (Columns AP-AS) reveals that the simulated genotype frequencies closely match theoretical expectations, verifying that the simulation accurately models inheritance patterns without introducing bias.

### 3.3. The Fate of the Neighboring Loci Under No POH Selection

Examining the fate of nine neighboring loci simulated to be on the same haplotype without recombination, some of them zeroed out and became fixed typically between generations 100–200, while the rest of the markers, maintaining their heterozygosity, reached the other upper extreme (Figure S3A). When we considered the nine neighboring loci with cM distances from 5 to 45 (M1 = 5 cM, M2 = 10 cM, M3 = 15 cM, M4 = 20 cM, M5 = 25 cM, M6 = 30 cM, M7 = 35 cM, M8 = 40 cM, M9 = 45 cM) allowing recombinations with distance-dependent probabilities (in our case, the recombination probability between M0 and any neighboring marker M1–M9 was ranging from 0.05 to 0.45 in 0.05 increments), the H_OBS_ values of all of these loci were uniformly zeroed sooner or later at different generation numbers in each iteration (Figure S3B).

### 3.4. POH Simulations with Two SNPs (H_OBS_)

Parameter conditions M2-BP3-Li4-Ge50. By using two SNPs, the POH selection (Figure S4A) follows essentially the same shape as seen with just one SNP locus (Figure 2). Here, the runs also strongly fluctuate; however, in this case, these are within narrower limits compared to that of the single-SNP model. Only one replicate (yellow curve) reached the H_OBS_ = 0.1 value, once. For the rest, the lowest value observed among the triplicates was above, or near to, the value of 0.4. The highest value observed was 0.82.

### 3.5. POH Simulations with Three SNPs (H_OBS_)

Parameter conditions M3-BP3-Li4-Ge50. In contrast to the previously presented M1 and M2 models, the amplitude of M3 ranged only between H_OBS_ = 0.4 and 0.8 (Figure S4B).

Increasing the number of considered markers from one to two, then to three SNPs, noticeably reduced the amplitude of the H_OBS_ value fluctuation. The comparison of SNP simulations with 1–2–3 markers illustrates that simulations using the POH model have an increased and maintained diversity in a controlled manner.

### 3.6. POH Simulations with 51 Unlinked SNPs (H_OBS_ and H_EXP_)

Parameter conditions: M51-BP(3,5,10)-Li4-Ge1000. In this section, we were interested in the sustainability of the diversity over an extremely long period of time (Ge = 1000), keeping the different BP values.

The BP3-Li4-Ge1000 simulation revealed that the H_OBS_ value eventually reached zero in the 289th generation (Figure 3). The BP5-Li4-Ge1000 simulation also led to decrease in the heterozygosity values, but they did not reach zero (H_OBS_ = 0.168 and H_EXP_ = 0.138 at F1000). On the other hand, the BP10-Li4-Ge1000 simulation did not show any loss of H_OBS_ and H_EXP_ values (Figure 3). The H_OBS_ values were above the initially observed 0.485 in all generations, and it was 0.528 at the generation of F1000. H_OBS_ values were above H_EXP_ in the cases of BP5 and BP10, as well.

### 3.7. Comparison of the POH Selection and ASp Method (H_OBS_ and H_EXP_)

Parameter conditions: M51-BP(5,10)-Li4-Ge1000. The values of H_OBS-POH_ and H_EXP-POH_ were above that of the H_OBS-ASp_ and H_EXP-ASp_ at BP5 and BP10. The H_EXP-POH_ values remained stationary at BP10.

At BP = 5 and the 1000th generation, the POH and ASp approaches declined in H_OBS_ values to 0.168 and 0.051, respectively (Figure 4A), representing a 3.3-fold increase over the ASp method. POH’s H_EXP_ value, at 0.139, was 3.5 times that of ASp. The ASp approach led to the decline of the original H_EXP-F0_ = 0.493 value observed in the starting (F0) population and the trend continued to the end of the 1000 generation simulation, where the H_EXP_ASp_ value was 0.040 (Figure 4B). At BP = 10 and the 1000th generation, the POH method demonstrated a H_OBS_ value of 0.528, exceeding the H_OBS-ASp_ = 0.297 value by a factor of 1.78. Similarly, POH’s H_EXP_ = 0.456 value was 1.79 times greater than ASp’s H_EXP_ = 0.254.

H_OBS_ and H_EXP_ values were explored from BP = 5 to 21 (Figure S5). A decline of H_EXP-POH_ values over the generation were noticed at BP = 9, while a decrease in H_EXP-ASp_ values was seen from BP = 15.

## 4. Discussion

The aims of our modeling were to evaluate the behavior of POH selection and to compare its characteristics with selection for minimal allele-sharing values of the parents. The model is an extreme case of avoidance of inbreeding, where genetic gain based on phenotypic value, quantitative trait loci, and genotype–environment interaction were not involved. It should be noted that our extremely reduced 51-SNP-set is just symbolic. Its major function is to promote simple simulation of the litters resulting from the matings.

The starting population for our calculations is a dog breed. It is undeniable that different evolutionary histories among organisms lead to varying linkage patterns, and dogs have their own characteristics. Since we wanted to introduce POH selection in its pure form, linkage disequilibrium (LD) among several other criteria, was not considered for incorporation into the model. Consequently, at that stage, the choice of the base population could have been any other diploid organism.

The reliable operation of the POH approach is illustrated in Figure 2. The fluctuating (‘zigzagging’) nature of the POH selection was most prominent when only one marker was used, and this setup allowed H_OBS_ to reach a value of 1.0. When additional markers were included in the model, the H_OBS_ = 1.0 value became inaccessible (see Section 3.4 and Section 3.5). The rhythmic, trend-like fluctuation of the curves between the extreme values appeared to be a regular, intrinsic feature of the model. The 1–2–3 SNP experiments, using only a few markers and extremely low-value parameter combinations, provided evidence that POH selection can maintain the genetic diversity of the populations at these loci; however, other loci not involved in the POH selection might lose their heterozygosity (Figure S3), as expected [1,2,24,25]. This supports the need for a denser marker set to be involved in the selection procedure.

When increasing the number of markers to 51, the SNP set included markers from both the proximal and the distal chromosomal regions on the longer (>50 cM) chromosomes. On the shorter (<50 cM) chromosomes, markers were selected only from the central areas. With this approach, a low genome coverage was achieved without the need to simulate genome-wide recombinations or consider haplotype blocks, which would be essential in the case of denser coverages. This marker set can be a minimal whole-genome set, suitable for demonstrating the experimental power of the POH selection at these loci. However, a much denser marker set is necessary for technical implementation in breeding practices.

Using the set of 51 markers, the amplitude range of heterozygosity narrowed substantially, which is congruent with the previously observed trend in the M1, M2, and M3 models. In summary, our results demonstrate that applying the POH-selection method can maintain genetic diversity with as few as ten breeding pairs, suggesting that endangered populations with a small number of breeding pairs might survive. The POH selection can sustain genetic diversity in the long term when the BP ≥ 10 and Li = 4.

Comparing the selection for the lowest number of shared alleles [13] to the POH selection on the same grounds, both approaches performed well at BP > 15. A conservation genomics strategy based on probability values (POH selection) appears to be more preferable when the number of breeding pairs is in the range of 5 ≤ BP ≤ 15 and can maintain the high diversity values over long periods. Taken together, the current study focused on demonstrating POH-selection performance using independent markers, and allowed us to determine its typical characteristics compared to ASp selections. The method can be extended to include additional genetic markers, and to simulate mutations, recombination, linkage disequilibrium, haplotypes, and more [26,27].

### Applicability

An important decision in animal breeding is the selection of mating pairs. Beyond the primary goals focused on production, it is crucial to avoid inbreeding and maintain genetic diversity, which are also major considerations in composing a mating plan, particularly for small and endangered populations. In the current genomic era, whole-genome polymorphism data from individuals can be effectively used to guide these decisions, helping to achieve a balance between production goals and genetic health [4,28].

The POH-selection method described here utilizes SNP information to maintain maximal heterozygosity across generations. However, some caution is needed when selection is based on maximal heterozygosity. Historically, breeds may have experienced events that introduced “foreign blood”, meaning registered individuals might have ancestors from other breeds. Such individuals could unintentionally be chosen as top breeders [17]. The POH selection should only be applied to individuals confirmed to belong to the breed in question [17,29]. This prefiltering enhances the reliability of the subsequent POH-selection step, aligning with the stringency requirements of the breeding community.

When the sole selection goal is to maintain and enhance genetic diversity, POH selection aligns with the general objectives of conservation breeding. However, implementing this method in practical breeding requires a much denser map, due to the potential loss of heterozygosity at unselected loci, as demonstrated in this article and by previous studies [1,2,24,25].

In species where the breeding plan differs from that of major domestic species, and where the prosperity of a breed relies on multiple stakeholders (such as breeders, clubs, government, etc.), POH selection can guide the overall breeding strategy. The management of dog breeds serves as a prime example of this model in action. Individual breeders have their own selection preferences, while government bodies and national or international breeding clubs may prioritize the maintenance of heterozygosity, particularly for endangered breeds with small population sizes.

These organizations might provide financial support for matings selected through the POH method, ranking them according to their conservation goals. This allows breeders to either optimize their breeding choices based on personal preferences, or adopt the personalized recommendations provided by the POH selection. Consequently, conservation-oriented organizations could significantly influence the conservation strategy within the selection framework of a particular breed [30,31].

## 5. Conclusions

We believe this study demonstrates that POH selection is a valuable method for maintaining genetic diversity, particularly in small or endangered populations with as few as ten breeding pairs. While a minimal set of markers was sufficient to illustrate the model’s effectiveness, a much denser marker set is necessary for practical application in breeding, to avoid the loss of heterozygosity at unselected loci. Compared to selection for minimal number of shared alleles, POH selection appears advantageous for populations with a very small number of breeding pairs. For the successful implementation of this method, it is worthwhile to carry out a genomic breed assignment test first, to reliably confirm the breed classification. POH selection offers a tool for conservation-focused breeding programs, allowing organizations to guide and support mating choices.

## Figures and Tables

**Table 1:** The list of the 51 SNP markers used for the simulations.

Chr	Name	Position	Chr	Name	Position
1	BICF2G630717404	31.39	14	TIGRP2P193757_rs9022836	44.02
1	BICF2P595364	96.65	15	BICF2P961117	53.01
2	BICF2P1055956	25.33	16	BICF2P356666	39.04
2	TIGRP2P33588_rs8522226	82.36	17	BICF2P1056918	44.38
3	G219f30S242	16.86	18	chr18_40029262	40.03
3	BICF2S23716752	85.01	19	BICF2P1128700	40.00
4	BICF2S23435937	20.37	20	BICF2P634022	27.01
4	BICF2P1161	81.94	21	BICF2P602990	41.08
5	BICF2S23660065	19.20	22	BICF2G630334870	47.96
5	BICF2S23623158	76.26	23	BICF2P738162	38.99
6	chr6_13131245	13.13	24	chr24_39910460	39.91
6	TIGRP2P89380_rs9117561	73.46	25	BICF2P695146	38.44
7	BICF2G630551940	13.19	26	BICF2S22941037	27.53
7	BICF2P217247	77.05	27	BICF2G630148964	22.54
8	BICF2S23545820	13.83	28	BICF2P1453414	30.49
8	BICF2S23625688	71.25	29	BICF2G630628668	29.06
9	BICF2G63024896	8.57	30	chr30_32985257	32.99
9	BICF2P562174	61.03	31	BICF2G630738767	25.41
10	BICF2P1125271	9.42	32	BICF2G630591581	21.00
10	TIGRP2P142790_rs9081297	68.94	33	TIGRP2P389175_rs8645908	23.02
11	BICF2G630292923	13.11	34	BICF2G630460012	25.37
11	BICF2S23322669	74.37	35	BICF2P930662	20.07
12	BICF2P831620	13.29	36	TIGRP2P414358_rs8929031	24.78
12	chr12_71954599	71.95	37	BICF2P1255908	26.12
13	BICF2G630606729	10.98	38	BICF2G63071428	16.52
13	BICF2S23043799	63.08			

Chr: chromosome number, name: marker name, position: location of the marker expressed in megabase.

**Table 2:** POH and ASp values of a locus depending on the genotypes of the parents.

Pairs	POH	ASp_(0–1)_	ASp
00	0	1	0.5
10 or 01	0.5	0.5	0.25
20 or 02	1	0	0
11	0.5	1	0.5
12 or 21	0.5	0.5	0.25
22	0	1	0.5

## Data Availability

The original contributions presented in this study are included in the article. Further inquiries can be directed to the corresponding author.

## Supplementary Materials

The following supporting information can be downloaded at: https://www.mdpi.com/article/10.3390/ani15152217/s1, Figure S1: Observed heterozygosities (Hobs) of the generations in the case of POH (A) and random selection (B). The number of markers is one, the number of breeding pairs is three. Part A is from Figure 1(A1–A3). Generation: subsequent pools of offspring generated from the selected pairs of the previous population. Different colors are representing different simulations; Figure S2: Figure 2 in Excel format. Simulations with one SNP. A1–A3: plots of H_OBS_ values in POH selection, B: steps of simulation with 1 SNP at generations F41-47. Hobs: observed heterozygosity. Generation: subsequent pools of offspring generated from the selected pairs of the previous population. Under the enlarged part of A1 the selection steps for these generations at the individual level are shown. Yellow highlight is the POH value of the offsprings of a given pair. AA, AB, and BB: genotypes of a locus. #obs and #exp: observed and expected number of AA, AB, and BB individuals. Hobs: observed heterozygosity, Hexp: expected heterozygosity. H_EXP_ and H_OBS_ values are outlined by providing bold borderlines of the corresponding cells; Figure S3: POH selection of one SNP (M0) and nine neighboring loci (M1–M9) with 5-10-15-20-25-30-35-40-45 cM distances without recombination (A) and with distance-dependent recombination (B). Generation: subsequent pools of offspring generated from the selected pairs of the previous population. Different colors are representing different markers (M0–M9) in the simulation; Figure S4: POH simulations with two (A) and three (B) SNPs. BP: number of breeding pairs. Generation: subsequent pools of offspring generated from the selected pairs of the previous population. Different colors in section A and B are representing different simulations; Figure S5: Observed and expected heterozygosity (Hobs and Hexp) of POH and ASp selections at different number of breeding pairs. BP: number of breeding pairs. Generation: subsequent pools of offspring generated from the selected pairs of the previous population. Different colors are representing simulations with different number of breeding pairs. Each line was obtained by averaging the values of 50 simulations.

## Abbreviations

The following abbreviations are used in this manuscript:

POH	Probability of the Offspring Heterozygosity
SNP	Single Nucleotide Polymorphism
LD	Linkage Disequilibrium
M	Marker/SNP number
BP	number of Breeding Pairs
Li	number of offspring per litter
Ge	number of simulated generations
ASp	Proportion of shared alleles
