# Supplementary material for: A Novel Conservation Genomic Strategy: Selection for the Probability of Offspring Heterozygosity
Source: Animals (Basel). 2025 Jul 28;15(15):2217. doi: 10.3390/ani15152217 (PMC12345495; doi:10.3390/ani15152217)
Supplement: Supplementary file 1 [file animals-15-02217-s001.zip › Figures_S1_S3_S4_S5_new.pdf]

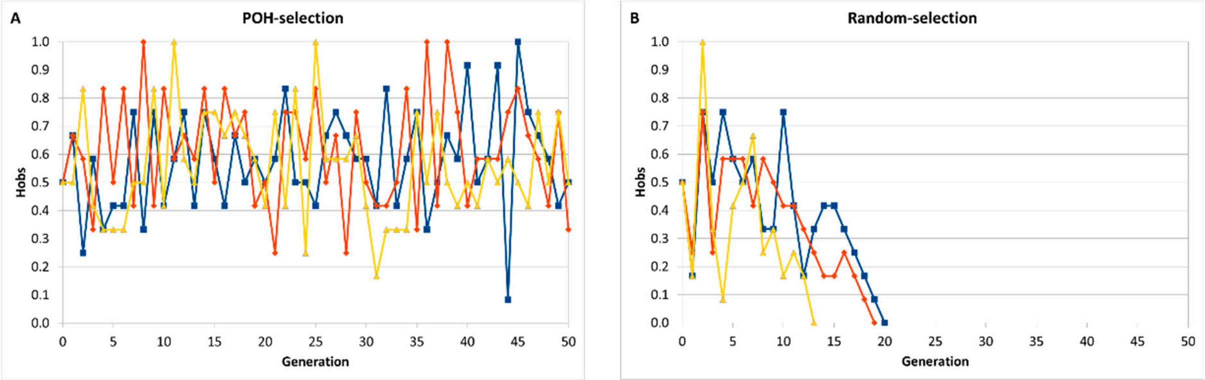

Figure S1. Observed heterozygosities (Hobs) of the generations in case of POH (A) and random selection (B). The number of marker is one, the number of breeding pairs is three. Part A is from Figure 1A1-3**.** Generation: subsequent pools of offspring generated from the selected pairs of the previous population. Different colors are representing different simulations

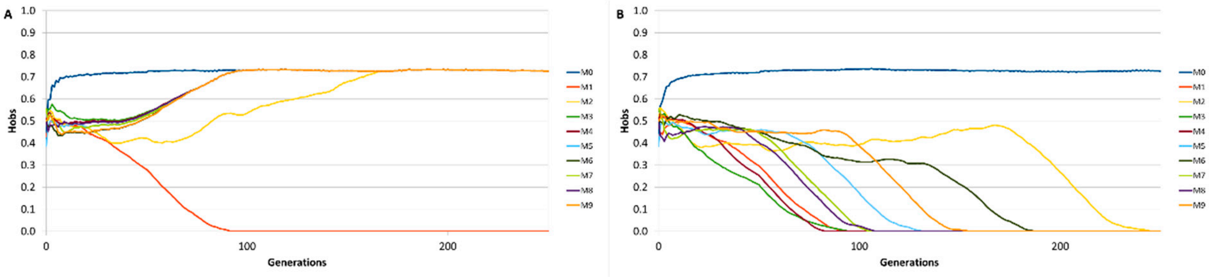

Figure S3. POH selection of one SNP (M0) and nine neighboring loci (M1-M9) with 5-10-15-20-25-30-35-40-45 cM distances without recombination (A) and with distance dependent recombination (B). Generation: subsequent pools of offspring generated from the selected pairs of the previous population. Different colors are representing different markers (M0-M9) in the simulation.

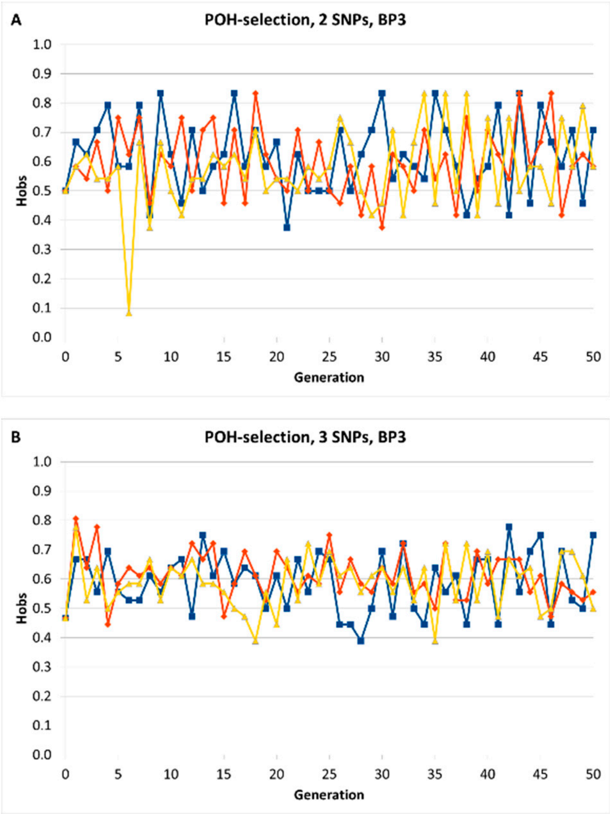

Figure S4. POH simulations with two (A) and three (B) SNPs. BP: number of breeding pairs. Generation: subsequent pools of offspring generated from the selected pairs of the previous population. Different colors in section A and B are representing different simulations

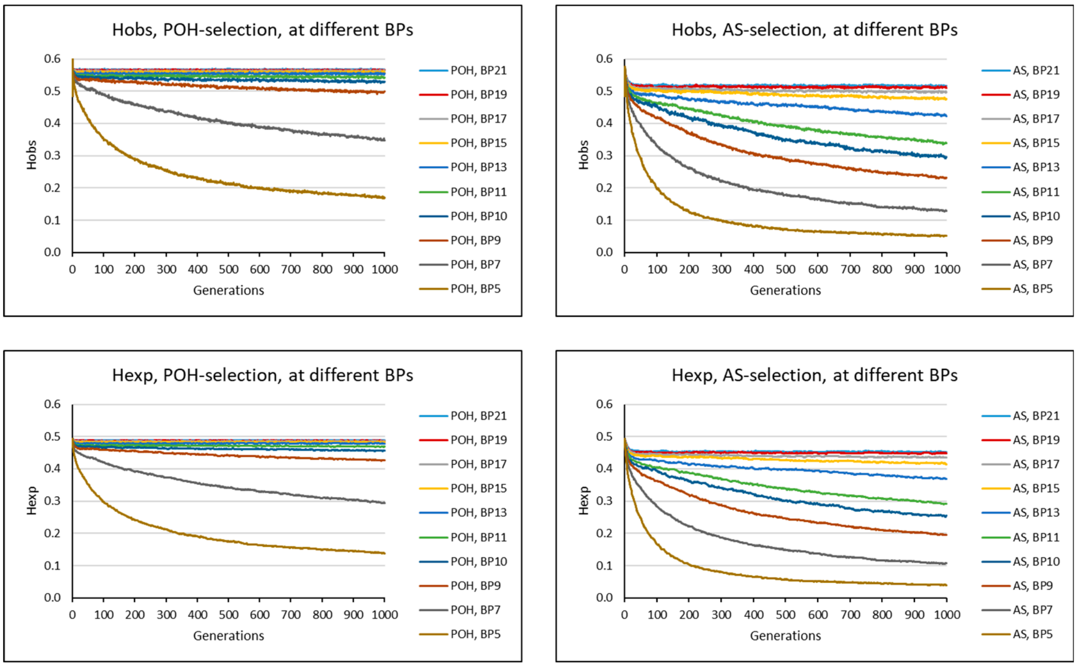

Figure S5. Observed and expected heterozygosity (Hobs and Hexp) of POH- and ASp-selections at different number of breeding pairs. . BP: number of breeding pairs. Generation: subsequent pools of offspring generated from the selected pairs of the previous population. Different colors are representing simulations with different number of breeding pairs. Each line was obtained by averaging the values of 50 simulations.
